# A hidden history of heartburn: The milk-alkali syndrome

**DOI:** 10.4103/0253-7613.75677

**Published:** 2011-02

**Authors:** Krishnan Swaminathan

**Affiliations:** Consultant Physician & Endocrinologist, Ward 14, Victoria Hospital, Kirkcaldy KY2 5AH,

**Keywords:** Antacids, hypercalcemia, renal failure

## Abstract

Milk-alkali syndrome was once considered to be of historic interest and a rare cause of hypercalcemia. Currently, it should be an important consideration in the differential diagnosis of hypercalcemia, after malignancies and primary hyperparathyroidism. The resurgence is in part due to the easy availability of over the counter (OTC) calcium preparations. We describe a 50-year-old man who presented with severe hypercalcemia on two occasions associated with renal failure and metabolic alkalosis. Extensive investigations during the first admission failed to unravel a specific cause of hypercalcemia but a thorough history during his subsequent admission helped to confirm the diagnosis of milk-alkali syndrome.

## Case Report

A 50-year-old man was referred by his general practitioner with a 5-week history of lethargy, nausea, polyuria and polydipsia. There was no significant past history or family history and clinical examination was non-contributory. Blood investigations revealed marked hypercalcemia of 3.5 mmol/L (2.15–2.65 mmol/L), urea 19.4 mmol/L (3–8.5 mmol/L), creatinine 425 *μ*mol/L (50–120 *μ*mol/L), estimated glomerular filtration rate (eGFR) 13 mL/min and venous bicarbonate of 36 mmol/L (22–32 mmol/L). Subsequent investigations revealed a suppressed parathyroid hormone (PTH) <8 ng/L (8–55 ng/L), a normal chest radiograph and protein electrophoresis. In view of the suppressed PTH levels and hypercalcemia, a wide variety of PTH-independent causes of hypercalcemia were considered. This included malignancy, sarcoidosis vitamin D, drug induced like lithium use), and milk-alkali syndrome.

A detailed history revealed previous admission 2 years ago, in a different hospital with similar symptoms and biochemistry. Investigations during that admission showed a normal computed tomography (CT) study of the chest and abdomen, bone scan and sestamibi scan of the parathyroids. The patient’s condition had improved with rehydration though the exact diagnosis was not made. Further, the patient was admitted with severe heartburn for 6 weeks preceding this admission for which he had self-medicated with excessive amounts of Rennie’s tablets (calcium carbonate 680 mg and magnesium carbonate 80 mg). Therefore, a diagnosis of milk-alkali syndrome was considered due to the combination of excess alkali ingestion, hypercalcemia, renal failure, associated metabolic alkalosis and exclusion of other causes of PTH-independent hypercalcemia.

The patient was treated with prompt rehydration and the antacid tablets were discontinued. This led to a gradual recovery of calcium and renal function. A proton pump inhibitor was prescribed and the patient was educated regarding the dangers of prolonged and excessive ingestion of over the counter (OTC) antacids. He maintained normocalcemia and normal renal function on a subsequent out-patient follow-up in 6 weeks.

## Discussion

In 1915, Sippy devised a calcium laden regime for treating peptic ulcer disease.[1] By 1936, a syndrome of hypercalcemia, renal failure and alkalosis was described.[2] As no alternatives existed, this therapy was popular till the advent of histamine receptor antagonists in the 1970s. However, there has been resurgence in this disorder in recent times[3] due to the availability of OTC calcium preparations and the emphasis on calcium therapy for prevention of osteoporosis.

The exact pathogenesis of milk-alkali syndrome is not completely understood. The key feature relates to a reduction in the renal ability to excrete excess calcium in susceptible individuals. A decrease in the glomerular filtration rate and excess tubular reabsorption of calcium due to metabolic alkalosis lead an increase in serum calcium. This leads to a suppression of serum PTH levels. In circumstances of continued ingestion of excessive calcium and alkali, significant complications including nephrocalcinosis, metastatic calcifications and progressive irreversible renal failure can occur.[4,5]

In this case, a thorough history and corroborative biochemistry not only helped to arrive at the diagnosis but also saved the patient from undergoing unnecessary investigations during this admission. Early identification and treatment of this disorder could prevent further progression to chronic, irreversible renal failure. Though the modern management of peptic ulcer disease has radically changed over the past decades, milk-alkali syndrome may still occur, especially in patients who self-medicate for symptoms of dyspepsia.
